# From clinical remission to biological stability in psoriasis: an antigen–blood–tissue framework for relapse stratification

**DOI:** 10.3389/fimmu.2026.1875991

**Published:** 2026-09-07

**Authors:** Zeyun Qiao, Lei Tang, Nana Luo, Zilin Cheng, Pingsheng Hao

**Affiliations:** 1 Clinical Medical College, Chengdu University of Traditional Chinese Medicine, Chengdu, Sichuan, China; 2 Department of Dermatology, Hospital of Chengdu University of Traditional Chinese Medicine, Chengdu, Sichuan, China; 3 Shanxi University of Chinese Medicine, Taiyuan, Shanxi, China; 4 Department of Dermatology and Cosmetology, Chongqing Traditional Chinese Medicine Hospital, Chongqing, China

**Keywords:** biological remission, biomarkers, inflammatory memory, psoriasis, relapse, tissue-resident memory T cells, treatment withdrawal

## Abstract

Relapse after treatment withdrawal remains a major challenge in psoriasis. Despite high rates of clinical clearance with modern systemic therapies, durable off-therapy control is uncommon, and recurrence often reappears at previously affected sites. Current models centered on resolved-skin inflammatory memory explain important aspects of local recall, but may not fully account for the heterogeneity of relapse timing, pattern and durability seen across patients. Here, we review psoriasis relapse through a clinically oriented multilevel framework that integrates three interacting dimensions: an antigenic dimension, blood-accessible relapse-associated signals and programs, and tissue permissiveness. Presentation-level antigen evidence raises the possibility that selected psoriatic phenotypes are associated with distinct upstream inflammatory programs, although direct links to relapse remain unproven. Circulating skin-homing memory populations, relapse-associated blood candidates and resident-derived mobile programs suggest, but do not establish, that relapse-relevant memory may remain accessible outside the skin during remission. At the tissue level, molecular scarring, epidermal tissue-resident memory T cells and stromal–epithelial support help explain why recurrence is preferentially re-executed in previously involved skin. We propose the Antigen–Blood–Tissue model as a falsifiable structure for interpreting relapse heterogeneity rather than a closed explanation of recurrence. Clinically, this framework suggests that visible clearance and biological stability should not be considered interchangeable. It also supports the view that remission may represent a biologically variable state, and that prospective studies aligned to treatment tapering or withdrawal will be needed to determine whether biological stability can eventually be defined beyond clinical remission and related prospectively to relapse outcomes.

## Highlights

- Clinical clearance in psoriasis does not necessarily indicate biological stability.
- This review presents a clinically oriented multilevel framework in which psoriasis relapse is examined across an antigenic dimension, blood-accessible relapse-associated signals and programs, and tissue permissiveness.
- The proposed framework provides a basis for testing whether biological stability can be defined beyond clinical remission.

## 1 Introduction: why current psoriasis relapse models remain incomplete

Psoriasis relapse after treatment withdrawal remains a major unresolved clinical problem despite the transformative efficacy of modern systemic therapies (1, 2). Many patients achieve complete or near-complete skin clearance, yet durable off-therapy stability is uncommon, and recurrence frequently reappears at previously affected sites (3). These observations have substantially reshaped current thinking by showing that clinically resolved psoriasis is not immunologically normal. Instead, healed skin may retain residual inflammatory programs, persistent immune memory, and stromal or epithelial alterations that together preserve local recall competence even after visible lesions have disappeared (4, 5).

This framework has advanced the field considerably, particularly by explaining why relapse often displays marked site fidelity and why “clear skin” does not necessarily indicate true biological reset. However, when viewed through a clinical rather than purely mechanistic lens, important gaps remain. Patients with apparently similar levels of clinical clearance can differ substantially in the timing, durability, and pattern of recurrence after treatment withdrawal (6, 7). Relapse-free intervals vary across therapeutic classes, repeated withdrawal may shorten the interval to recurrence, and emerging prediction studies suggest that rebound liability is not entirely stochastic (8). These features indicate that resolved-skin inflammatory memory, although central, may not by itself fully explain relapse dynamics.

Existing relapse frameworks address different parts of this problem. Resolved-skin inflammatory memory and TRM-centred models explain persistent local recall competence and site fidelity, whereas studies of circulating skin-homing memory and tissue-to-blood exchange suggest that disease-relevant immune programs may remain available beyond the skin. These perspectives are complementary, but they do not explicitly separate three questions that are often discussed together: what may organize inflammatory directionality, how relapse-relevant memory remains accessible during remission, and where that memory is converted into visible recurrence.

The Antigen–Blood–Tissue (ABT) framework does not replace established models of resolved-skin memory or systemic immune activation. Rather, it organizes relapse-related observations into three biologically distinct dimensions. The antigenic dimension concerns whether selected disease trajectories retain reproducible upstream immunological specificity; the blood dimension concerns relapse-associated cellular and soluble signals that can be followed during remission without assuming that peripheral blood itself is the site of long-lived pathogenic memory; and tissue permissiveness concerns residual immune, epithelial, and stromal features that may facilitate local recall in previously involved skin. The framework is intended to examine whether considering these dimensions together helps explain differences in relapse timing, anatomical pattern, and durability, while allowing their relative contribution and evidentiary support to differ between patients. The sections that follow review both the evidence and the limitations of each dimension. Unless otherwise stated, the discussion focuses on chronic plaque psoriasis that has achieved clinical clearance after systemic treatment, because most available evidence on treatment withdrawal, resolved-skin memory, and relapse-associated biomarkers has been generated in this setting.

## 2 Antigenic dimension: from psoriasis pathogenesis to relapse hypotheses

### 2.1 From candidate lists to presentation-level evidence

For many years, psoriasis was interpreted predominantly through the lens of cytokine dysregulation, with putative autoantigens discussed largely as upstream possibilities rather than experimentally resolved drivers (9, 10). This emphasis was understandable, as the therapeutic success of IL-17- and IL-23-directed agents strongly reinforced the view that psoriasis is a cytokine-amplified inflammatory disease (11). However, a cytokine-centric formulation alone does not fully explain why genetically predisposed individuals develop distinct clinical phenotypes, why certain inflammatory programs preferentially stabilize in particular tissue contexts, or why recurrence may not be entirely antigen-agnostic (12–14). These unresolved questions have renewed interest in the antigenic layer of disease, particularly in ways that move beyond speculative candidate lists.

A key advance in this regard is the emergence of immunopeptidomics, which allows naturally presented HLA-bound ligands to be measured directly in lesional tissue (14–16). This is an important shift in evidentiary level. Rather than inferring antigenicity indirectly from genetic association, transcript abundance, or T-cell reactivity alone, immunopeptidomic approaches begin to define what is actually displayed to T cells within the diseased microenvironment (14, 15). In psoriasis, this creates a more mechanistically coherent bridge between HLA-associated susceptibility, tissue-specific ligand presentation, and adaptive immune selection (9, 13, 17). At a conceptual level, it moves the field from asking whether psoriasis may involve antigen-specific responses to asking which responses are presented, in which context, and with what implications for disease organization.

This transition does not mean that psoriasis relapse has already been reduced to a defined antigen code, nor that all clinically relevant heterogeneity can be mapped neatly onto single antigenic axes. Presentation-level evidence may eventually contribute to measurable endotyping, but only if antigen–phenotype associations prove reproducible across cohorts and remain informative over time (14, 15). At present, however, immunopeptidomics remains primarily a discovery-stage approach that requires tissue sampling and specialized mass spectrometry, while HLA genotyping and antigen-reactive T-cell assays provide only indirect or technically demanding complementary readouts and lack validated thresholds for relapse prediction. Whether such associations predict relapse after treatment withdrawal is currently unknown. The immediate value of antigen discovery is therefore to generate biologically anchored candidate subsets for prospective testing, rather than to assign patients to established antigen-defined relapse categories. In this sense, examples such as SERPINB3 are treated here as hypothesis-generating endotype anchors, not as validated relapse classifiers.

### 2.2 SERPINB3 as a phenotype-linked example of antigen presentation

An instructive example of how presentation-level evidence can inform psoriasis endotyping comes from the overlap spectrum between psoriasis and atopic dermatitis (18, 19). Although these diseases have traditionally been framed as immunologically distinct, a clinically and histologically recognizable intermediate zone exists in which psoriasiform and eczematous features coexist or alternate over time (20). This overlap is not simply a descriptive curiosity. Rather, it suggests that at least some psoriatic presentations may be organized by antigenic and inflammatory programs that are more specific than a generic “psoriasis cytokine state” would imply (21, 22). In this setting, the concept of an antigen-linked endotype becomes especially useful, because it offers a way to connect phenotype, tissue context, and adaptive immune recognition within a single interpretive framework.

SERPINB3 offers a useful but preliminary proof-of-principle example. Recent immunopeptidomic work identified naturally presented SERPINB3-derived ligands in psoriasis, with the strongest enrichment in eczematized psoriasis rather than conventional plaque disease (14). This finding links antigen presentation to a recognizable inflammatory phenotype and moves beyond candidate-autoantigen cataloguing (23). However, the observation is phenotype-specific, has not yet been independently reproduced, and was not designed to examine treatment withdrawal or subsequent relapse. It therefore supports the feasibility of antigen-informed stratification, but does not establish a clinically meaningful relapse endotype. In other words, SERPINB3 is valuable not simply because it adds one more putative target to the psoriasis literature, but because it illustrates how presentation-level evidence can begin to anchor an experimentally tractable disease subset (14, 22, 23).

Importantly, SERPINB3 should be interpreted as a proof-of-principle rather than as a universal template for all psoriasis relapse biology. Its potential value as an endotype marker will require independent replication in broader psoriasis cohorts and longitudinal assessment across treatment response, remission, and relapse. Until such evidence is available, the finding should not be interpreted as a clinically validated marker of relapse risk. Current evidence does not justify reducing disease heterogeneity to a single antigenic pathway, nor does it establish that all relapse-prone states are directly antigen-defined. The broader significance of this example lies elsewhere: it shows that antigen presentation can, under specific tissue and cytokine conditions, align with a clinically meaningful phenotype strongly enough to support endotype-level reasoning (14, 22, 24). Once that becomes possible, psoriasis can be viewed less as a uniformly relapsing inflammatory disorder and more as a family of partially distinct relapse-prone states, some of which may be more tightly anchored to defined antigenic circuits than others (24). This is precisely the type of shift needed if relapse heterogeneity is to be understood in a way that is both mechanistically grounded and prospectively testable.

### 2.3 Antigenic niches and the problem of relapse heterogeneity

Antigenic information is unlikely to be clinically meaningful if it is interpreted only as a list of putative targets. In psoriasis, antigens are not encountered by the immune system in an abstract or uniform manner; rather, they are selected, processed, licensed, and amplified within local microenvironments that determine whether a potentially relevant ligand remains immunologically silent or becomes integrated into a self-sustaining inflammatory circuit (13, 26, 27). For this reason, antigen biology is best understood in terms of antigenic niches rather than isolated antigen identities. Here, an antigenic niche is narrower than the broader inflammatory microenvironment: it refers specifically to the local combination of antigen source and availability, processing and presentation machinery, HLA or CD1 context, and tissue-derived co-signals that allows antigen-specific immune recognition to become pathogenic. General inflammatory cues may support this niche, but they do not constitute an antigenic niche unless they influence antigen availability, presentation, or antigen-specific recall (27). This framework is particularly useful for relapse biology because it offers a way to explain why antigen-linked responses may stabilize differently across patients and why some recurrent trajectories may be more tightly coupled to specific tissue contexts than others.

The LL-37 axis illustrates this principle well. LL-37 is not simply an abundant antimicrobial peptide that happens to be immunogenic; its relevance emerges within a niche created by barrier stress, keratinocyte activation, innate danger signaling, and local cytokine amplification (25, 28, 29). In this setting, antigen supply is coupled to an inflammatory environment that promotes immune activation rather than tolerance. The consequence is not merely recognition of a target, but the formation of a repeatable stress-to-effector loop in which tissue injury, innate licensing, and adaptive recall reinforce one another (30). Read in this way, the LL-37 pathway is informative not because it identifies a single dominant psoriasis antigen, but because it shows how a tissue-conditioned niche can make an antigenic circuit biologically durable and therefore potentially relapse-relevant.

A second example is provided by the HLA-C*06:02-linked melanocyte axis, including ADAMTSL5-associated responses (13, 17). Here, genetic susceptibility, target-cell geography, and antigen presentation are aligned more tightly than in many other proposed psoriasis pathways (9). The importance of this niche lies not only in the existence of a recognizable target, but in the fact that antigen display is embedded within a restricted anatomical and genetic context that can favor selective immune engagement. Antigen processing pathways may further tune this circuit, reinforcing the idea that presentation is not fixed but biologically regulated (31). In niche terms, this behaves less like a diffuse inflammatory tendency and more like a partially locked interface between genetic risk, cellular localization, and antigen-directed immune recognition. Such a configuration provides a plausible basis for why some forms of psoriasis may show more stable, phenotype-linked relapse behavior than would be expected under a purely cytokine-defined model.

Antigenic niches also extend beyond classical peptide–HLA interactions (26). Nonclassical routes, including lipid-related antigen presentation, indicate that the relevant question is not simply which molecule is recognized, but which local conditions make that recognition pathogenic (32). Likewise, keratinocytes need not be framed as primary priming antigen-presenting cells to remain highly important in this architecture (27). Even when they do not initiate adaptive responses, they may still act as “last-mile” amplifiers by sustaining antigen availability, inflammatory crosstalk, and permissive recall conditions within previously affected skin. This broader view is important because it prevents antigen biology from being interpreted too narrowly. What matters for relapse is not only the existence of a candidate target, but whether the tissue environment repeatedly converts that target into an executable inflammatory program.

These antigen-related pathways are supported by different types and levels of evidence and should not be treated as equivalent. LL-37 is supported by evidence of T-cell autoreactivity, nucleic acid-dependent inflammatory amplification, and increased expression in active lesions that declines following treatment (25, 28–30, 65). The ADAMTSL5–HLA-C*06:02 axis has a more restricted genetic and anatomical basis, with evidence involving melanocyte localization, ERAP1-dependent peptide generation, and T-cell receptor recognition (13, 17, 31). However, neither pathway has been prospectively linked to relapse after treatment withdrawal. SERPINB3 is supported by presentation-level and functional evidence, but mainly in eczematized psoriasis rather than conventional plaque psoriasis or relapse cohorts (14, 23). CD1a-dependent lipid presentation represents a distinct nonclassical route of autoreactivity, although its contribution during remission and recurrence remains unknown (26, 32). These findings therefore support several antigen-informed disease configurations rather than a single antigen-defined relapse pathway.

For relapse-oriented interpretation, however, the level of evidence needs to be kept clear. Demonstrating that an antigen participates in psoriasis pathogenesis does not show that the same pathway persists during remission or contributes to later recurrence. Current evidence for LL-37 and the ADAMTSL5–HLA-C*06:02 pathway comes mainly from mechanistic and disease-associated studies in active psoriasis, whereas SERPINB3 adds presentation-level and phenotype-linked evidence, primarily in eczematized psoriasis. CD1a-dependent lipid presentation similarly supports a nonclassical route of autoreactivity. What remains largely unknown is whether any of these antigen-specific programs persist during clinical remission, track with subsequent relapse, or improve prospective prediction after treatment withdrawal. None of the antigen pathways discussed here has yet been prospectively validated as a predictor of relapse timing or remission durability, and direct evidence that antigen-specific pathways causally govern recurrence is lacking. The antigen component of ABT is therefore best regarded as a hypothesis-generating upstream dimension rather than an established determinant of relapse.

Seen through this niche-based lens, distinct antigen pathways converge on a common implication: relapse heterogeneity may reflect differences not only in downstream cytokine intensity, but also in how specific tissue contexts select and stabilize particular immunological routes back into disease (13, 26, 27). This does not mean that all recurrent psoriasis can already be mapped onto discrete antigen-defined categories. However, it does mean that antigenic directionality is most plausibly linked to relapse when it is embedded within a local niche capable of preserving pathogenic logic through remission. In that sense, antigenic niches provide the conceptual bridge between presentation-level evidence and the broader question of why relapse-prone psoriasis is heterogeneous rather than immunologically uniform.

## 3 Beyond resolved-skin memory: blood-accessible relapse-associated signals and programs

Resolved-skin memory provides a strong explanation for local recall competence, but relapse-associated activity may also be detectable outside the skin during remission and treatment withdrawal (2, 4, 35). Peripheral blood is therefore useful as an accessible compartment for longitudinal assessment of circulating cells and soluble mediators that may reflect residual or re-emerging disease activity (5, 34).What can be measured in blood, however, should be distinguished from the anatomical compartment in which long-lived pathogenic memory is maintained. Several biologically distinct observations contribute to this blood-accessible dimension in psoriasis: circulating CLA^+^ skin-homing populations, relapse-associated cellular subsets, soluble blood-phase signals, and evidence from broader inflammatory settings that some resident-like T-cell programs can re-enter the circulation (34–37). These findings do not necessarily have the same biological meaning. Circulating cells may be transiently trafficking, may derive from cutaneous or lymphoid compartments, or may themselves participate in renewed inflammation. Soluble mediators are better interpreted as inflammatory readouts or biomarkers, whereas circulating TRM-like populations currently provide mainly a possible link to tissue-to-blood exchange.

### 3.1 CLA^+^skin-homing memory in circulation

One cellular component of the blood-accessible dimension is the circulating CLA^+^ memory compartment. CLA identifies T cells with preferential skin-homing potential and therefore provides an accessible blood-based window into immune programs with direct relevance to cutaneous disease (38, 39). In psoriasis, circulating CLA^+^ T-cell populations have long been linked to skin-directed inflammation, and treatment-responsive fluctuations in their blood frequency are consistent with the idea that skin-directed immune activity remains systemically represented even when plaques improve clinically (35, 40–42). This matters because a relapse model based exclusively on resident tissue archives risks overlooking the systemic availability of cells that remain capable of re-entering skin under permissive conditions.

The value of circulating CLA^+^ memory lies not only in its skin-homing potential but also in its accessibility for longitudinal study. These cells provide a measurable representation of skin-directed immune activity outside the skin and may therefore help track how such activity changes during remission and recurrence (34, 43, 44). Their presence in blood, however, does not establish where long-lived pathogenic memory is maintained or whether these cells directly initiate relapse. The circulating CLA^+^ pool is therefore better viewed as an accessible cellular population for studying relapse-associated biology than as evidence that peripheral blood itself functions as a memory reservoir (42).

### 3.2 Relapse-associated circulating candidates

A second and more specific layer of evidence comes from the identification of circulating candidate subsets that appear enriched in relapse-prone disease states. This is important because the CLA^+^ compartment is not immunologically uniform. If relapse biology is genuinely blood-accessible, then some subsets should carry a more direct relationship to recurrence than others. Recent work identifying circulating CLA^+^CTSW^+^ cytotoxic CD4^+^ populations is therefore particularly notable, as it suggests that remission may harbor measurable blood programs associated with earlier relapse after biologic withdrawal rather than being immunologically silent (33). Such findings shift the discussion from general plausibility to testable biological candidates.

The recent CLA^+^CTSW^+^CD4^+^ study compared peripheral blood collected at biologic withdrawal from patients who subsequently experienced early or late relapse and combined single-cell profiling with assessment of CTSW in psoriatic lesions and cell–cell communication analysis (33). It therefore provides evidence extending beyond a simple cross-sectional change in cell frequency. However, CTSW was not functionally perturbed, the cohort was not stratified by biologic class, and the findings have not yet been independently reproduced. The subset should therefore be regarded as one recent relapse-associated blood candidate rather than as the defining basis of a circulating reservoir. The blood layer rests more broadly on the combined evidence from CLA^+^ skin-homing biology, disease-associated circulating programs, and soluble blood-phase signals, none of which alone establishes causality.

The significance of these emerging subsets lies less in their current status as biomarkers than in the questions they raise. Cellular and soluble blood signals also need to be distinguished biologically. Circulating CLA^+^CTSW^+^CD4^+^ cells represent a cellular program whose role in relapse remains uncertain, whereas serum IL-19 is more appropriately interpreted as a soluble inflammatory readout. Changes in IL-19 around treatment withdrawal and clinical recurrence may therefore have value for longitudinal monitoring (45, 46), but they do not identify where relapse-relevant immune memory is maintained. Their biological role, however, remains unresolved. Such populations could act as drivers that return to the skin and participate in renewed inflammation, markers of residual or re-emerging systemic immune activity, or bystanders that track relapse without contributing directly to it. Existing studies do not distinguish among these possibilities, and direct longitudinal tracking from remission-phase blood to recurrent plaques within the same patients remains lacking. Resolving this question will require paired blood–skin sampling across treatment withdrawal and relapse, clonotype or lineage tracking, and functional studies of migration and effector activity. For now, these populations are best regarded as relapse-associated candidates rather than established causal mediators.

### 3.3 ex-TRM and tissue-to-blood exchange

Evidence that resident-like T-cell programs can re-enter the circulation provides a possible mechanism of tissue-to-blood exchange (36, 37, 47). Tissue-resident memory T cells have traditionally been defined by long-term persistence within peripheral tissues, and their importance in site-biased psoriasis recurrence is strongly implicated (48, 49). However, increasing data across inflammatory settings suggest that at least some resident-like cells can re-enter circulation, giving rise to ex-TRM or circulating TRM-like populations that retain features of prior tissue residence while recovering systemic mobility (36, 37, 47). This possibility is conceptually relevant to psoriasis because it offers a potential mechanism through which local inflammatory history might remain systemically accessible after lesion resolution.

In relapse terms, tissue-to-blood exchange offers one possible way to reconcile site-biased recurrence with longitudinal systemic continuity. However, this remains biological plausibility rather than demonstrated psoriasis relapse biology. Most supporting evidence comes from non-psoriatic settings, and definitive lineage-resolved tracking from resolved psoriatic skin to blood and then to recurrent plaques is lacking. Circulating TRM-like cells may also represent phenotypically similar circulating memory cells rather than cells directly derived from previously lesional skin. Accordingly, ex-TRM are retained in the ABT framework only as a hypothesis-generating mechanism of tissue-to-blood exchange, while the blood dimension rests primarily on psoriasis-specific observations from circulating CLA^+^ populations and other relapse-associated blood signals.

## 4 Tissue permissiveness: where relapse is executed

Even if relapse-associated activity is detectable beyond the skin, recurrence is not random. Psoriatic lesions tend to return to preferred sites, indicating that previously affected skin retains a permissive architecture for recall long after visible inflammation has subsided. Clinically resolved psoriasis is therefore not biologically equivalent to never-lesional skin. Residual molecular programs may persist within healed plaques, creating an intermediate state in which inflammatory circuitry has been suppressed but not fully dismantled (5, 50). This helps explain why relapse is often better understood as reactivation of incompletely resolved disease biology rather than as *de novo* inflammatory construction (2).

A central component of this permissive state is the persistence of local immune memory. Epidermal tissue-resident memory T cells remain one of the most plausible cellular archives of relapse competence because they combine long-term tissue persistence with rapid effector potential. Once established within previously involved skin, these cells can lower the threshold for renewed inflammation when permissive conditions return (48, 49). More recent single-cell profiling during IL-23 blockade further supports the persistence and treatment-responsive remodeling of skin-resident pathogenic T-cell programs, reinforcing the view that local resident memory remains a major component of the relapse-permissive niche (51). Their importance lies less in explaining every feature of relapse than in explaining why recurrence often shows marked site fidelity. In this sense, epidermal TRM help define where psoriasis remembers how to return, even if they do not fully determine when relapse occurs or how durable remission will be across individuals.

Tissue permissiveness also extends beyond resident lymphocytes. Keratinocytes may retain residual stress programs, altered inflammatory responsiveness, and barrier-related instability even after clinical improvement, making previously lesional skin easier to re-engage in pathogenic crosstalk (5, 50). Fibroblast states may likewise contribute by sustaining positioning signals and tissue contexts that remain relatively favorable to renewed immune activation (4). These epithelial and stromal elements do not replace immune memory; rather, they influence how efficiently retained pathogenic programs are converted into visible plaques. Their relevance is therefore best understood in terms of execution rather than initiation: they help shape whether a given burden of relapse-relevant memory becomes clinically manifest at a specific site.

Taken together, molecular scarring, epidermal TRM, and stromal–epithelial support are best viewed as interconnected components of a local recall niche. Within the ABT framework, this niche acts as a local threshold modifier rather than as an independent source of relapse or the final step of a fixed linear sequence. Similar circulating immune activity or antigen-linked inflammatory input may remain clinically silent in non-permissive skin but be converted into recurrence where residual TRM, keratinocyte stress programs, and stromal positioning cues lower the threshold for reactivation. Conversely, a permissive tissue state may not produce relapse without sufficient upstream input. Thus, circulating memory may help explain how relapse competence remains available during remission, while tissue permissiveness helps determine whether and where that competence becomes a visible plaque. Resolved-skin inflammatory memory therefore remains indispensable within the broader framework, not because it is sufficient on its own, but because it conditions the local conversion of retained pathogenic programs into site-specific recurrence.

## 5 A falsifiable antigen–blood–tissue model for relapse stratification

**Figure 1:**
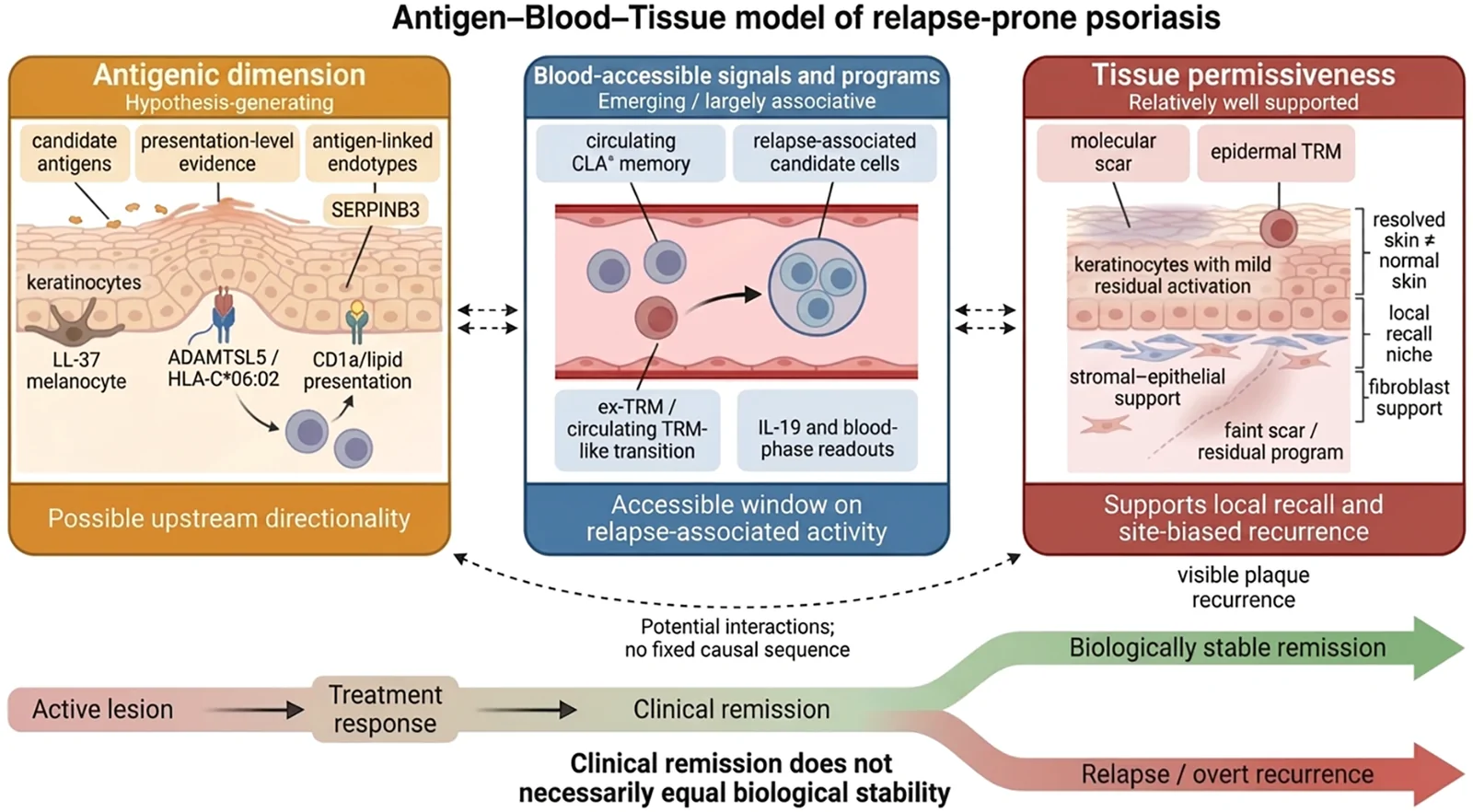
Antigen–blood–tissue model of relapse-prone psoriasis.

Taken together, the preceding evidence supports examining psoriasis relapse across three biologically distinct dimensions rather than as a fixed sequential pathway (52). The antigenic dimension is the most tentative and asks whether some disease trajectories retain reproducible upstream immunological specificity (14, 25, 53, 54). The blood-accessible dimension comprises circulating cellular programs and soluble signals associated with relapse-related activity during remission and treatment withdrawal (7, 55). It concerns what can be observed in peripheral blood rather than assigning long-lived pathogenic memory to the blood compartment itself. Tissue permissiveness refers to residual immune, epithelial, and stromal conditions in previously involved skin that may support local recall and site-biased recurrence (5, 49, 50). These dimensions are not proposed as successive steps, and their relative contribution may vary between patients. The ABT model therefore serves as a conceptual and falsifiable framework for asking whether information across these dimensions helps explain variation in relapse timing, anatomical pattern, and durability, rather than as a quantitatively validated prediction model or clinical decision tool (**Figure 1**).

Existing concepts explain different parts of psoriasis relapse. Resolved-skin inflammatory memory and TRM-centred models provide a strong basis for local persistence and site-biased recurrence, whereas systemic immune activation provides a context for inflammatory activity that remains detectable in the circulation. Antigen-centred studies address a different question, namely whether selected disease trajectories may retain some degree of upstream immunological specificity. The ABT model does not treat any of these observations as unique to the framework. Its added value instead depends on whether these dimensions provide information that is not fully redundant during remission, and whether considering them together helps explain variation in relapse beyond local tissue memory alone. In this sense, ABT separates local recall competence, systemically accessible relapse-associated activity, and possible antigen-related directionality while allowing their relative contribution to differ between patients (56, 57).

The evidentiary basis of the three dimensions is not equivalent. Tissue permissiveness is supported most directly by psoriasis-specific studies of resolved lesions and resident memory, whereas evidence for blood-accessible relapse-associated activity remains largely associative. The antigenic dimension is more preliminary still. Current studies support antigen-related mechanisms and phenotype-linked presentation in psoriasis, but provide little direct evidence for persistence during remission, prospective relapse prediction, or causal involvement in recurrence. Antigen-related biology is therefore treated here as a hypothesis-generating upstream dimension rather than an established relapse determinant. The three dimensions should not therefore be interpreted as carrying equal evidentiary weight, a distinction that is also apparent in the available longitudinal and treatment-withdrawal studies summarized in **Table 1**. Direct relapse evidence is concentrated in clinical withdrawal outcomes and a limited number of blood- and tissue-based studies, whereas the antigenic dimension is still supported mainly by pathogenesis and phenotype-linked evidence rather than longitudinal relapse data. Alternative interpretations also remain possible: circulating subsets may be markers rather than drivers, and presented antigens may identify inflammatory states without determining relapse. The ABT framework is therefore used to formulate testable relationships, not to imply a validated causal sequence.

**Table 1:** Representative longitudinal and treatment-withdrawal studies relevant to psoriasis relapse.

Study	Therapy/withdrawal design	Relapse definition/time	Compartment & candidate	Longitudinal/pre-relapse?	Independent replication	Evidence level/key finding
Gallais Sérézal et al., 2018 (48)	NB-UVB; resolved-lesion sampling after treatment, then prospective follow-up	Clinical recurrence during 6–18 months; earlier relapse linked to the tissue response	Resolved skin; IL-17/IFN-related inflammatory response	Single post-treatment biopsy; yes, sampled before subsequent relapse	No; exploratory cohort (n=9)	Prospective tissue association with ex vivo functional assessment; direct pre-relapse signal, not causal proof
VOYAGE 2: Reich et al., 2017; Gordon et al., 2019 (66, 67)	Guselkumab (IL-23p19 blockade); week-28 PASI90 responders randomized to withdrawal or maintenance	Loss of PASI90; median 15.2 weeks after withdrawal randomization (~23 weeks after last dose)	Serum; IL-17A, IL-17F, IL-22, IL-23	Serial serum sampling; no—cytokine rebound lagged behind PASI worsening	No independent cohort; biomarker analysis nested in VOYAGE 2	Randomized withdrawal plus associative pharmacodynamic evidence; limited relapse-prediction value
Harris et al., 2021 (PAUSE) (45)	Ustekinumab induction; abatacept vs continued ustekinumab, followed by withdrawal	Loss of ≥50% of initial PASI improvement; median ~36 vs 32 weeks from last ustekinumab dose	Skin & serum; lesional transcriptome and IL-19/cytokines	Serial weeks 0/12/24/40; molecular re-emergence observed, but preclinical lead not established	No independent withdrawal cohort	Randomized mechanistic withdrawal study; temporal biomarkers remain associative
Huang et al., 2024 (6)	Multiple biologics; 12-year, six-center real-world withdrawal cohort (991 treatment episodes)	Clinical relapse after biologic discontinuation; time-to-relapse modeled across therapies	Clinical variables only; no biological candidate	Longitudinal clinical follow-up; biological pre-relapse sampling not applicable	Multicenter cohort; no external biological validation	Large real-world clinical association; treatment and withdrawal history predict durability, not mechanism
Chiu et al., 2025 (33)	Biologic withdrawal; prospective relapse classification after discontinuation	Subsequent clinical relapse during follow-up; timing used to classify relapse outcome	PBMCs; CTSW+ relapse-associated T-cell population	Single sample at withdrawal; yes, measured before outcome classification	No independent cohort or functional CTSW validation	Prospective blood association; candidate pre-relapse cellular signal, not mechanistic proof

NB-UVB, narrowband ultraviolet B; PASI, Psoriasis Area and Severity Index; PBMC, peripheral blood mononuclear cell.

The present configuration is intended primarily for chronic plaque psoriasis achieving clinical clearance after systemic treatment, because most available evidence on treatment withdrawal, resolved-skin memory, and relapse-associated blood signals has been generated in this setting. It should not be generalized without qualification to guttate or pustular psoriasis, in which trigger and immune configurations may differ, or to psoriatic arthritis, where joint and entheseal compartments must also be considered. These phenotypes may therefore require different weighting or adaptation of the antigen, blood, and tissue components, and subtype-specific validation remains necessary.

Relapse may also be precipitated by external perturbations that are not themselves a fourth ABT layer but may act on one or more of its components. Infection may alter antigen availability, innate immune activation, and circulating skin-directed responses, whereas mechanical trauma may disrupt barrier integrity and lower the local threshold for reactivation. Psychological stress and seasonal variation may likewise modify systemic inflammatory tone or tissue permissiveness. The clinical effect of these exposures is therefore likely to depend on the pre-existing configuration of antigen-related biology, blood-accessible relapse-associated activity, and local tissue state. The same trigger may remain clinically silent in one patient or site but precipitate recurrence in another, and some relapses may occur without an identifiable external trigger.

Treatment itself also complicates the interpretation of relapse after withdrawal. Pharmacokinetic half-life, duration of target suppression, tissue exposure, pharmacodynamic persistence, treatment duration, and mechanism of action can all influence the interval between the last dose and visible recurrence. These treatment-related effects are better regarded as modifiers of observed time to relapse than as a fourth ABT dimension. A prolonged relapse-free interval may reflect greater biological stability, persistent pharmacological suppression, or both. Time to relapse should therefore not be used as a direct proxy for residual ABT-associated biology without considering treatment exposure and the expected persistence of drug effect.

The value of the ABT model depends on whether it generates predictions that extend beyond any one of its components. The antigenic dimension remains the most tentative. Current evidence supports the involvement of antigen-related pathways in psoriasis pathogenesis and in phenotype-linked presentation contexts (14, 25, 31). If this dimension is also relevant to relapse stratification, however, reproducible antigen-presentation patterns should be associated with subsequent relapse phenotype or trajectory, rather than simply distinguish active psoriasis from non-lesional or healthy skin. For the blood dimension, relapse-associated activity should add information about subsequent recurrence in at least some patients with comparable treatment exposure and similar residual tissue-memory features. Existing withdrawal-aligned studies make this possibility worth testing, but do not yet show that blood-based measures provide information beyond established tissue features (33, 45). Finally, if local tissue permissiveness influences whether systemic relapse-associated activity is converted into visible inflammation, similar blood-phase activity should not necessarily lead to the same pattern of recurrence across anatomical sites. The known tendency of psoriasis to recur at previously involved sites is consistent with this possibility and provides a clinically relevant setting in which to test it (56, 57). The model therefore does not require all three dimensions to contribute equally in every patient; its added value depends on whether at least some cross-layer information proves informative beyond tissue memory alone.

The ABT framework should also be open to findings that argue against its added value. If, after accounting for treatment-related effects, relapse timing and anatomical recurrence could be explained adequately by features of resolved skin alone, and blood- or antigen-related measures consistently failed to add reproducible information, the rationale for a multilevel model would be substantially weakened. The blood component would also be called into question if longitudinal circulating signals showed no consistent relationship with subsequent relapse beyond overall disease activity and treatment exposure. Likewise, the antigenic component would have limited relevance to relapse stratification if presentation patterns were not reproducible across cohorts or over time, or if they remained unrelated to subsequent relapse phenotype or trajectory. More generally, if combining information across the ABT dimensions did not improve explanation or prediction beyond tissue-based measures, the central claim that a multilevel framework adds value would not be supported.

## 6 Clinical translation: toward a research definition of biological remission

The main clinical implication of this framework is that surface clearance and biological stability should no longer be treated as interchangeable endpoints (58, 59). In this review, biological remission is treated as a proposed research construct rather than a currently measurable disease state. It is intended to capture the possibility that patients with similar clinical clearance may retain different degrees and patterns of relapse-associated biology. What would define such a state remains uncertain: the relevant blood and tissue measures, the appropriate reference baseline, the magnitude and duration of change required, and the relative weighting of different biological compartments have not been established. Persistent TRM, or any other single residual feature, should therefore not by itself be used to define or exclude biological remission. A patient may achieve complete or near-complete resolution of visible plaques while still retaining relapse-relevant tissue archives, blood-accessible relapse-associated activity, or both (2, 4, 60). This distinction matters because the current language of remission in psoriasis is still weighted toward what can be seen clinically, whereas relapse biology is increasingly suggesting that disease persistence may continue beneath the threshold of overt inflammation. In this sense, remission is better understood not simply as an absence of plaques, but as a transitional biological state whose underlying stability may vary substantially between patients (58). The translational implications of this framework are summarized in **Table 2**.

**Table 2:** Translational summary of the antigen–blood–tissue framework in psoriasis relapse.

Layer	Core concept	Representative readouts/evidence	Clinical relevance
Antigenic dimension	Possible upstream immunological directionality; hypothesis-generating in relapse	Candidate antigens; presentation-level evidence; LL-37; ADAMTSL5/HLA-C*06:02; SERPINB3	Supports hypothesis-driven testing of phenotype-linked relapse biology
Blood-accessible signals and programs	Circulating cellular and soluble indicators of relapse-associated activity during remission	Cellular candidates: circulating CLA^+^ memory and relapse-associated subsets; soluble readouts: IL-19 and related biomarkers; tissue-to-blood mobility: circulating TRM-like populations	Supports longitudinal blood-based assessment while separating monitoring signals from the anatomical source of long-lived memory
Tissue permissiveness	Local conditions that may support local recall and site-biased recurrence	Molecular scar; epidermal TRM; keratinocyte residual programs; stromal–epithelial support	Helps explain site-biased recurrence and local recall competence
Integrated clinical implication	Interaction among antigenic, circulatory and tissue-level layers	Combined interpretation of endotyping, blood-phase signals and selected resolved-skin features	Supports prospective testing of biological stability beyond clinical clearance

CLA, cutaneous lymphocyte-associated antigen; TRM, tissue-resident memory T cells; ex-TRM, resident-derived cells that regain systemic mobility.

This shift in perspective is especially relevant when treatment intensity is reduced or withdrawn (61–63). At present, the central question is often framed in binary terms: whether relapse will or will not occur after de-escalation. However, a biologically informed model suggests that the more useful question may be whether latent instability can be recognized before full clinical recurrence emerges (59). That possibility depends on moving beyond a purely skin-surface definition of control and toward a layered assessment of residual disease biology. Under such a model, patients with similar clinical responses may still differ in rebound liability according to the degree of antigenic organization underlying their disease, the persistence of blood-accessible relapse programs, and the permissiveness of previously affected tissue to reactivation (6, 60, 61).

Separating residual disease biology from residual treatment effect will require prospective withdrawal studies to interpret biological measurements in the context of treatment exposure. Serial changes should be considered alongside the timing of the last dose, expected pharmacokinetic and pharmacodynamic persistence, treatment duration, and mechanism of action. A signal that emerges only as drug effects wane may have a different meaning from one that remains detectable while target suppression is still expected to persist. Differences in time to relapse across therapies should therefore not be attributed to biological stability alone without accounting for major treatment-related differences.

A practical consequence is that remission may become a monitoring window rather than a passive waiting period (55, 61). At present, the ABT model is intended to guide prospective research rather than define biological remission or direct treatment decisions. Initial testing could combine characterization of active disease with serial blood sampling during stable remission, treatment tapering, or withdrawal, together with selected assessment of previously lesional skin where feasible. Candidate blood measurements may include circulating CLA^+^ memory populations, relapse-associated subsets, and soluble inflammatory signals, whereas tissue assessment may examine residual TRM and epithelial or stromal programs. These measurements should be related prospectively to relapse timing, anatomical distribution, and severity. No validated biomarker combination, detection threshold, or intervention trigger currently exists; these parameters would require derivation and external validation before the model could inform clinical decisions. Blood is especially attractive in this regard because it is accessible, repeatable, and more suitable than serial biopsies for longitudinal assessment. Circulating skin-homing memory pools, candidate relapse-associated subsets, and related molecular readouts could, in principle, be combined with selected tissue-based markers to define a more rigorous state of biological control (33, 46, 58). Such an approach would not require blood to replace skin, nor would it assume that one marker set will fit all patients. Rather, it points toward a stratified strategy in which blood-first assessment is complemented, where necessary, by tissue-level information from resolved lesions. The goal is not to overmedicalize remission, but to recognize that clinical quiescence may conceal different degrees of residual relapse competence.

This also has implications for how future studies should be designed. Prospective cohorts aligned to tapering, interval extension, or treatment withdrawal may provide the most informative setting in which to test whether biological remission can be distinguished from clinical remission in operational terms (62–64). Ideally, such studies would integrate active-lesion endotyping, remission-phase blood profiling, and selected assessment of previously involved skin, then relate these measurements to the timing, pattern, and severity of recurrence. If successful, this approach could move psoriasis management beyond the current emphasis on suppressing visible inflammation toward a more durable form of disease control, one in which the aim is not only to clear lesions, but also to reduce the biological likelihood of relapse.

## 7 Conclusion

Psoriasis relapse after treatment withdrawal may reflect the interaction of antigen-linked inflammatory directionality, blood-accessible relapse-associated activity, and a local tissue state capable of converting retained immune programs into recurrent plaques. The ABT model organizes these dimensions as a working, testable account of relapse heterogeneity rather than as a validated causal or clinical prediction model. Prospective studies combining active-disease characterization, serial remission-phase blood assessment, and selected analysis of previously lesional skin will be required to determine whether these dimensions improve prediction of relapse timing, pattern, and durability.
